# Association between prognostic nutritional index and diabetic retinopathy among U.S. diabetic adults in NHANES

**DOI:** 10.1038/s41598-025-96582-7

**Published:** 2025-04-15

**Authors:** Lizhen Xu, Xiling Lin, Ting Li, Junping Wen, Gang Chen

**Affiliations:** Department of Endocrinology, Shengli Clinical Medical College of Fujian Medical University, Fujian Provincial Hospital, Fuzhou University Affiliated Provincial Hospital, Fuzhou, 350001 China

**Keywords:** Diabetic retinopathy, Prognostic nutritional index, National health and nutrition examination survey, Endocrine system and metabolic diseases, Eye diseases, Endocrinology, Nephrology

## Abstract

Diabetic retinopathy (DR) is characterized by progressive retinal vascular damage that ultimately causes vision loss. The prognostic nutritional index (PNI), which integrates albumin and lymphocytes, serves as an indicator of an individual’s inflammatory response, nutritional condition, and immune system function. This research aimed to explore the possible association between PNI and DR. This was a cross-sectional study utilizing data from the National Health and Nutrition Examination Survey (NHANES) between 2001 and 2018. Weighted logistic regression analyses were employed to assess the relationship between PNI and DR prevalence. A total of 4791 adults aged 20 years and older were included in the analysis. Results indicated a statistically significant negative correlation between PNI and DR prevalence. In the fully adjusted model, a one-unit rise in PNI corresponded to a 7% reduction in the probability of DR prevalence. Quartile analysis consistently indicated that individuals in the highest PNI quartile had notably lower odds of DR prevalence compared to those in the lowest quartile. Additionally, smooth curve fitting suggested a nonlinear relationship between PNI and DR. Subgroup analysis reinforced the strength of the inverse association between PNI and DR (all *p* for interaction > 0.05). This nationally representative study demonstrated a significant inverse relationship between PNI levels and DR prevalence among diabetic adults in the United States. Our findings emphasize the potential role of maintaining optimal PNI values in preventing the development of DR.

**Supplementary Information:**

The online version contains supplementary material available at 10.1038/s41598-025-96582-7.

## Introduction

Diabetic retinopathy (DR), frequently observed as a complication of diabetes, involves the gradual deterioration of blood vessels within the retina, ultimately culminating in the loss of vision^1^. It has become a primary cause of visual impairment^2^. In 2019, the International Diabetes Federation reported that diabetes affected 463 million people globally, with projections indicating a potential increase to 700 million by 2045^3^.Studies have shown that nearly one-third of those with diabetes worldwide suffer from DR^4^. With the increasing prevalence of diabetes, the associated impact of diabetic retinopathy (DR) is anticipated to grow substantially in the years ahead^1^.Consequently, DR has become a critical global public health issue, highlighting the importance of identifying modifiable risk factors to manage and prevent DR effectively.

The prognostic nutritional index (PNI) serves as a straightforward and reliable indicator to evaluate an individual’s nutritional health, immune system activity, and inflammatory state. Its calculation is derived from measurements of serum albumin concentration and lymphocyte count^5^. Initially introduced in 1984, PNI was first applied to evaluate cancer prognosis^6^. Since then, growing evidence has demonstrated its utility across a wide range of health conditions, including predicting outcomes in colorectal cancer^7^, hepatocellular carcinoma and cervical cancer^8^, mortality in type 2 diabetes mellitus (T2DM) patients^9^, migraine^10^, albuminuria^5^. The growing range of clinical uses for PNI has attracted considerable attention in the medical community, emphasizing its significance as a promising biomarker for integration into standard clinical procedures.

Existing studies have highlighted a connection between higher PNI levels and a reduced prevalence of diabetic kidney disease^9^. Additionally, a cohort study revealed that serum albumin is associated with diabetic chronic microvascular complications, emphasizing its potential role in both the development and management of diabetes-related conditions^11^. Despite these findings, there remains a paucity of research exploring the direct association between PNI and DR. A cross-sectional investigation conducted on Indian T2DM patients offered preliminary evidence regarding the relationship between PNI and DR^12^. However, the study’s insights were constrained due to its single-center design and limited participant size, underscoring the necessity of broader, more diverse research to substantiate these findings. In response to this research gap, the current study examined the correlation between PNI and DR in a US-based diabetic population using data sourced from the National Health and Nutrition Examination Survey (NHANES). The aim was to deepen the understanding of the correlation between PNI and the prevalence of DR, with the goal of providing meaningful clinical insights and practical guidance for the treatment and management of DR.

## Materials and methods

### Study population

The National Center for Health Statistics (NCHS) in the USA developed the NHANES to perform a nationally representative cross-sectional survey, gathering insights on Americans’ nutrition and health for effective health management and early detection of emerging public health issues. To ensure a representative sample of the non-institutionalized civilian population across the United States, participants were selected through a sophisticated, stratified, multistage probability cluster sampling approach. Ethical oversight for the NHANES study was provided by the Ethics Review Committee of NCHS, which approved the study protocol. Prior to enrollment, all participants signed written informed consent forms. This investigation concentrated on individuals aged 20 and above, utilizing data spanning NHANES cycles from 2001 to 2018.

**Fig. 1:**
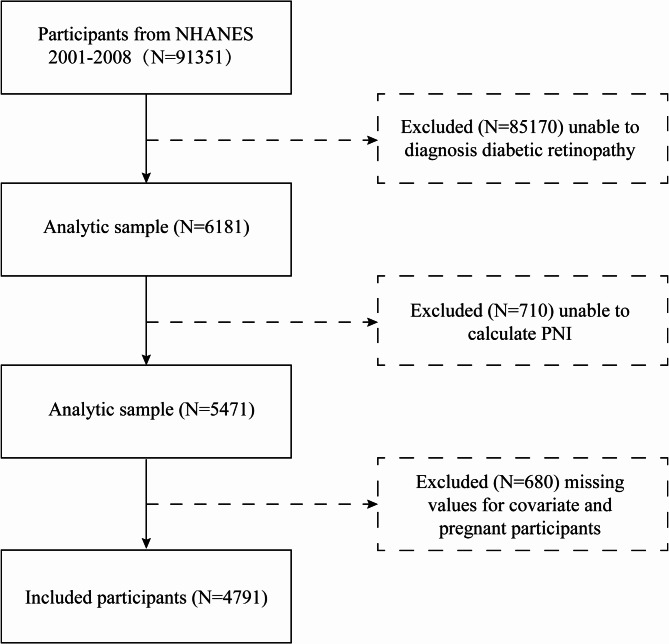
The flow diagram of the study participants, from NHANES 2001–2018.

Initially, 91,351 eligible participants were recruited. However, several exclusions were applied: 85,170 participants who could not complete a diabetic retinopathy evaluation, 710 with missing PNI data, and 680 either lacking full covariate information or identified as pregnant. Following these exclusions, the final analysis incorporated 4791 participants who satisfied all study criteria (Fig. 1).

### Exposure variables and outcome definition

In this study, PNI was employed as the primary exposure variable and computed using the formula: PNI = 10 × albumin (g/dL) + 0.005 × absolute lymphocyte count (10^3^cells/µL). To further reflect the dose-response relationship between PNI and DR, PNI was divided into four quartiles. Serum albumin levels were determined through the bromocresol purple dye method, while absolute lymphocyte counts were measured using a Beckman Coulter DxH 800 analyzer.

Diabetes mellitus was identified if one of the following criteria was satisfied: (1) glycohemoglobin levels ≥ 6.5%; (2) fasting glucose concentration ≥ 7.0 mmol/L; or (3) a self-reported prior diabetes diagnosis from a physician. Participants were labeled as having DR if they responded affirmatively to the question: ‘Has a doctor ever told you that diabetes has affected your eyes or that you had retinopathy?’.

### Covariates

Variables were selected based on established confounding factors identified in previous research and medical practices. To more accurately assess the relationship between PNI and diabetic retinopathy, the analysis included the following covariates: age (categorized as 20–40, ≥ 40), gender, race, marital status, education level, smoking status, hypertension, alcohol consumption, poverty-to-income ratio (PIR), healthy eating index-2015 (HEI-2015), physical activity level, last time had pupils dilated for exam, diabetic medications, obesity, total calorie intake, diabetic nephropathy (DN). HEI-2015 is an index used to measure whether the dietary habits of the U.S. population align with the Dietary Guidelines for Americans, with higher scores indicating healthier dietary habits^13^. The formula for calculating weekly physical activity time is the time spent in moderate-intensity physical activity plus twice the time spent in high-intensity physical activity, and participants with weekly physical activity time ≥ 150 min are considered physically active^14,15^. Obesity is defined as a body mass index ≥ 30 kg/m². Participants with a urinary albumin creatinine ratio ≥ 30 mg/g are considered to have DN^16,17^.

### Statistical analysis

This study adhered to the NHANES guidelines for statistical analysis, applying survey weights to address the intricacies of the sampling structure. Participant characteristics were summarized using descriptive statistics, categorized by the presence or absence of DR. Continuous variables were analyzed using a weighted linear regression model, whereas categorical variables were evaluated through a weighted chi-square test. To investigate the relationship between DR prevalence and PNI, weighted logistic regression analysis was performed. PNI was categorized into quartiles and the first quartile designated as the reference.

Three regression models were constructed to examine the data: Model 1 was an unadjusted crude model; Model 2 accounted for adjustments in age, gender, and race; Model 3 incorporated additional adjustments for marital status, education level, smoking behavior, hypertension, alcohol drinking, PIR, HEI-2015, physical activity level, last time had pupils dilated for exam, diabetic medications, obesity, total calorie intake, DN. To assess the potential non-linear relationship between PNI and the prevalence of DR, a smoothing curve fitting was incorporated into the fully adjusted analytical model.

Subgroup analyses, alongside interaction tests, were employed to investigate potential differences across various population groups. In the fully adjusted model, participants were categorized based on age, gender, race, marital status, education level, smoking behavior, hypertension, alcohol use, and PIR. To assess differences in effects across these subgroups, the interaction between PNI and potential modifying factors was analyzed (*p* for interaction).

We conducted two sensitivity analyses following established methodologies to validate result reliability. First, we re-examined the PNI-DR association using unweighted logistic regression, excluding adjustments for NHANES complex sampling design. Second, after excluding participants with extreme PNI values (< 5th or > 95th percentiles), we analyzed this relationship using both weighted and unweighted logistic regression models to ensure methodological consistency across different analytical frameworks.

All statistical analyses were performed utilizing the EmpowerStats software suite in conjunction with R software (version 4.2.0). The significance threshold for these analyses was established at a *P*-value of less than 0.05.

## Results

### Baseline characteristics

From 2001 to 2018, a total of 4791 participants were assessed for inclusion in the study. The average age of the participants was 61.95 ± 13.08 years, with males comprising 52.52% of the cohort, and the overall prevalence of diabetic retinopathy was 21.75%. As shown in Table 1, the PNI values of participants without DR were notably higher than those with DR (*P* < 0.01). Additionally, the DR group showed a higher prevalence of hypertension compared to the non-DR group (*P* < 0.01).

**Table 1:** Baseline characteristics of participants in NHANES 2001–2018 (*N* = 4791).

Characteristics	Non-diabetic retinopathy (*N* = 3749)	Diabetic retinopathy (*N* = 1042)	*P*-value
Age	61.89 ± 13.25	62.17 ± 12.45	0.543
Age(%)			0.071
20–40	236 (6.30%)	50 (4.80%)	
≥ 40	3513 (93.70%)	992 (95.20%)	
Gender(%)			0.239
Male	1952 (52.07%)	564 (54.13%)	
Female	1797 (47.93%)	478 (45.87%)	
Race(%)			0.091
Mexican American	743 (19.82%)	203 (19.48%)	
Other Hispanic	311 (8.30%)	101 (9.69%)	
Non-Hispanic White	1444 (38.52%)	363 (34.84%)	
Non-Hispanic Black	927 (24.73%)	266 (25.53%)	
Other Race	324 (8.64%)	109 (10.46%)	
Marital status(%)			0.312
Married	2125 (56.68%)	570 (54.70%)	
Widowed	555 (14.80%)	167 (16.03%)	
Divorced	492 (13.12%)	144 (13.82%)	
Separated	147 (3.92%)	35 (3.36%)	
Never married	312 (8.32%)	81 (7.77%)	
Living with partner	118 (3.15%)	45 (4.32%)	
Education status(%)			0.076
Less Than 9th Grade	619 (16.51%)	204 (19.19%)	
9-11th Grade	624 (16.64%)	179 (17.03%)	
High School Grad	882 (23.53%)	253 (23.90%)	
Some College or AA degree	1023 (27.29%)	256 (24.08%)	
College Graduate or above	601 (16.03%)	150 (14.11%)	
Smoking status(%)			0.281
YES	618 (16.48%)	153 (14.68%)	
NO	1308 (34.89%)	384 (36.85%)	
Other	1823 (48.63%)	505 (48.82%)	
Hypertension(%)			0.001
YES	2542 (67.80%)	761 (73.03%)	
NO	1201 (32.04%)	277 (26.58%)	
Other	6 (0.16%)	4 (0.38%)	
Alcohol drinking(%)			< 0.001
YES	972 (25.93%)	239 (22.94%)	
NO	816 (21.77%)	183 (17.56%)	
Other	1961 (52.31%)	620 (59.50%)	
Physical actibity level(%)			0.370
Inactive	679 (18.11%)	199 (19.10%)	
Active	1339 (35.72%)	348 (33.40%)	
Other	1731 (46.17%)	495 (47.50%)	
Last time had pupils dilated for exam(%)			0.875
Less than 1 month	29 (0.77%)	12 (1.15%)	
1–12 months	117 (3.12%)	33 (3.17%)	
13–24 months	38 (1.01%)	11 (1.06%)	
Greater than 2 years	45 (1.20%)	10 (0.96%)	
Never	19 (0.51%)	5 (0.48%)	
Other	3501 (93.38%)	971 (93.19%)	
Diabetic medications			0.277
YES	266 (7.10%)	79 (7.58%)	
NO	1005 (26.81%)	302 (28.98%)	
Other	2478 (66.10%)	661 (63.44%)	
Diabetic nephropathy			0.692
YES	340 (9.07%)	102 (9.79%)	
NO	2554 (68.12%)	697 (66.89%)	
Other	855 (22.81%)	243 (23.32%)	
Obesity			0.148
YES	851 (22.70%)	207 (19.87%)	
NO	2427 (64.74%)	698 (66.99%)	
Other	471 (12.56%)	137 (13.15%)	
Poverty-to-income ratio	2.31 ± 1.52	2.11 ± 1.49	< 0.001
Poverty-to-income ratio(%)			< 0.001
≤1	881 (23.50%)	282 (27.06%)	
1–3	1719 (45.85%)	500 (47.98%)	
>3	1149 (30.65%)	260 (24.95%)	
Healthy eating index-2015	53.80 ± 11.61	53.63 ± 11.65	0.677
Total calorie intake	1894.66 ± 785.21	1926.38 ± 854.13	0.258
Prognostic nutritional index	41.19 ± 3.50	40.29 ± 3.81	< 0.001

Continuous variables are expressed as Mean ± SD, with *P*-values determined using the weighted linear regression model; categorical variables are presented as (%), and the *P*-values were calculated using the weighted chi-square test.

### Association between PNI and DR

Weighted logistic regression analyses were conducted to explore the association between PNI and DR. As shown in Table 2, PNI was negatively associated with DR prevalence. Across all models, including the unadjusted model (Model 1, OR = 0.93, 95% CI: 0.91, 0.96, *p* < 0.001), the partially adjusted model (Model 2, OR = 0.93, 95%CI: 0.90, 0.95, *p* < 0.001), and the fully adjusted model (Model 3, OR = 0.93, 95%CI: 0.91, 0.95, *p* < 0.001), a consistent negative correlation was observed. The findings suggest that a one-unit rise in PNI corresponds to a 7% decrease in the prevalence of DR. Additionally, individuals in the highest PNI quartile (Q4) demonstrated a 53% reduced chance of developing DR relative to those in the lowest quartile (Q1)(Table 2, Model 3, OR = 0.47, 95%CI: 0.36, 0.62, *p* for trend < 0.001).

**Table 2:** Weighted logistic regression models for the association between prognostic nutritional index (PNI) and diabetic retinopathy (DR) in adults in the NHANES 2001–2018.

Characteristic	Model 1 OR (95%CI)	Model 2 OR (95%CI)	Model 3 OR (95%CI)
PNI	0.93(0.91,0.96)	0.93(0.90,0.95)	0.93(0.91,0.95)
Categories			
Q1	1.0	1.0	1.0
Q2	0.89 (0.71, 1.12)	0.89 (0.71, 1.12)	0.88 (0.70, 1.12)
Q3	0.63 (0.47, 0.85)	0.62 (0.46, 0.83)	0.63 (0.46, 0.84)
Q4	0.49 (0.38, 0.64)	0.47 (0.36, 0.61)	0.47 (0.36, 0.62)
*p* for trend	< 0.001	< 0.001	< 0.001

**Fig. 2:**
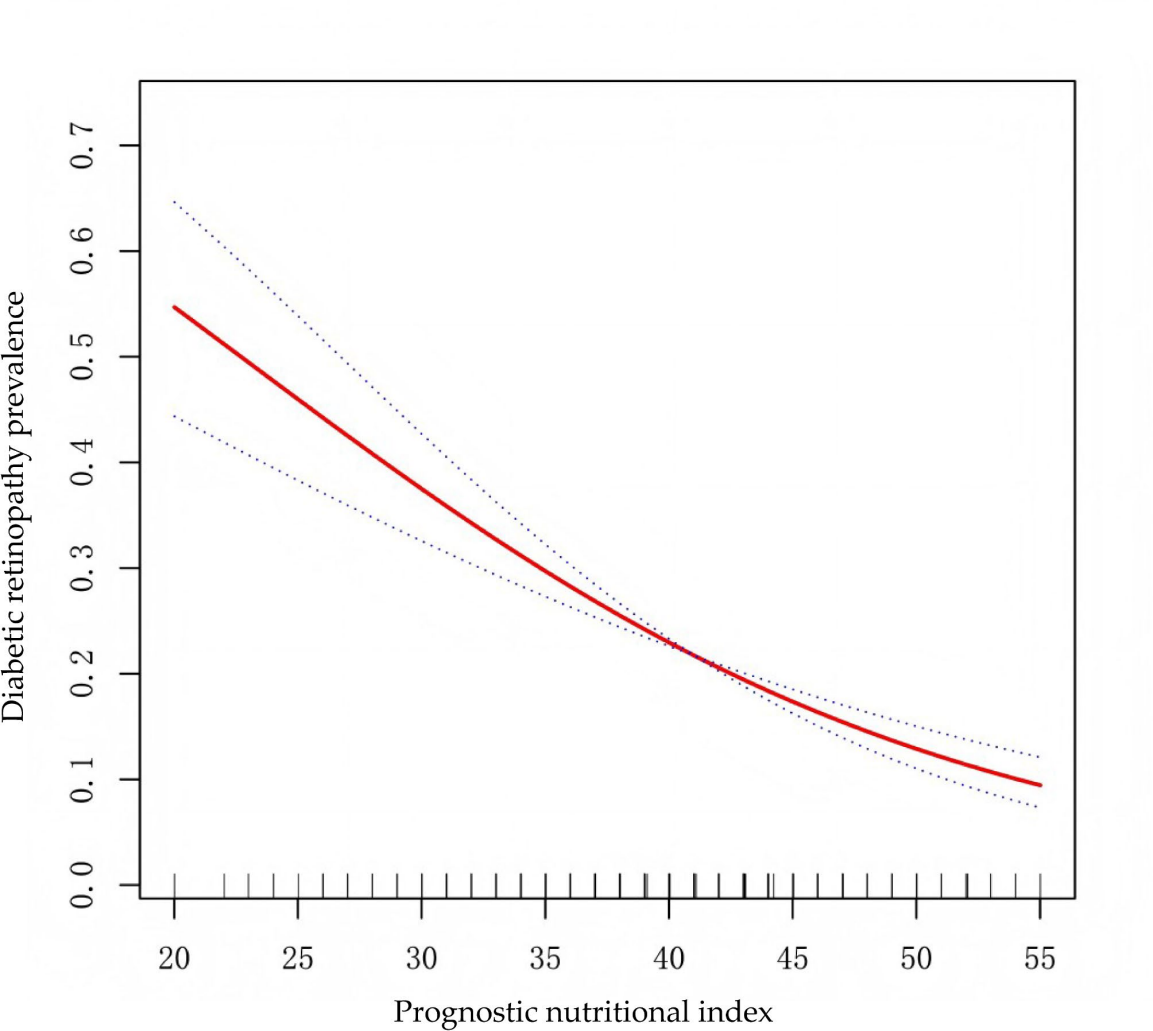
The nonlinear association between prognostic nutritional index and diabetic retionopathy prevalence.

Smoothed curve fitting, applied within the fully adjusted model, revealed further insights into the nonlinear relationship between PNI and DR (Fig. 2).

### Subgroup and sensitivity analyses

The PNI demonstrated a negative correlation with DR across all subgroups (all OR < 1). As shown in Table 3, subgroup analysis suggested that the negative correlation between PNI and DR was more pronounced among participants aged over 40 years (OR = 0.93, 95%CI: 0.90, 0.96, *p* < 0.001), female participants (OR = 0.92, 95%CI: 0.88, 0.95, *p* < 0.001), participants of Mexican American (OR = 0.90, 95%CI: 0.86, 0.95, *p* < 0.001), participants never married (OR = 0.90, 95%CI: 0.83, 0.98, *p* = 0.012), participants with hypertension (OR = 0.92, 95%CI: 0.90, 0.95, *p* < 0.001), non-drinking participants(OR = 0.88, 95%CI:0.82, 0.95, *p =* 0.001), participants with PIR ≤ 1 (OR = 0.91, 95%CI: 0.87, 0.95, *p* < 0.001), participants without DN (OR = 0.94, 95%CI: 0.91, 0.97, *p* < 0.001) .

**Table 3:** Stratified analysis of the correlation between prognostic nutritional index (PNI) and diabetic retinopathy (DR) in adults in the NHANES 2001–2018.

Subgroup	Model OR (95%CI)	*p*-value	*p* for interaction
Age			0.469
20–40	0.96(0.88,1.06)	0.459	
≥ 40	0.93(0.90,0.96)	< 0.001	
Gender			0.226
Male	0.94(0.91,0.98)	0.002	
Female	0.92(0.88,0.95)	< 0.001	
Race			0.673
Mexican American	0.90 (0.86, 0.95)	< 0.001	
Other Hispanic	0.92(0.85, 1.00)	0.065	
Non-Hispanic White	0.93 (0.90, 0.97)	< 0.001	
Non-Hispanic Black	0.93 (0.90, 0.96)	< 0.001	
Other Race	0.96 (0.89, 1.05)	0.374	
Marital status			0.896
Married	0.94 (0.90, 0.98)	0.001	
Widowed	0.93 (0.87, 0.98)	0.016	
Divorced	0.95 (0.89, 1.01)	0.116	
Separated	0.91 (0.81, 1.02)	0.101	
Never married	0.90 (0.83, 0.98)	0.012	
Living with partner	0.90 (0.78, 1.03)	0.134	
Education status			0.905
Less Than 9th Grade	0.94 (0.89, 0.99)	0.022	
9-11th Grade	0.94 (0.88, 1.01)	0.098	
High School Grad	0.94 (0.90, 0.99)	0.031	
Some College or AA degree	0.92 (0.88, 0.97)	< 0.001	
College Graduate or above	0.91 (0.85, 0.97)	0.007	
Smoking status			0.244
YES	0.95 (0.90, 1.00)	0.046	
NO	0.95 (0.91, 1.00)	0.039	
Other	0.91 (0.88, 0.95)	< 0.001	
Hypertension			0.307
YES	0.92 (0.90, 0.95)	< 0.001	
NO	0.96 (0.91, 1.01)	0.078	
Other	0.79 (0.55, 1.13)	0.194	
Alcohol drinking			0.162
YES	0.97 (0.91, 1.03)	0.260	
NO	0.88 (0.82, 0.95)	0.001	
Other	0.93(0.90, 0.96)	< 0.001	
Poverty-to-income ratio			0.178
≤ 1	0.91 (0.87, 0.95)	< 0.001	
1–3	0.95 (0.92, 0.98)	0.005	
> 3	0.93 (0.87, 0.98)	0.015	
Diabetic nephropathy			0.822
YES	0.94 (0.86,1.03)	0.206	
NO	0.94 (0.91, 0.97)	< 0.001	
Other	0.92 (0.86, 0.97)	0.005	

The results of two sensitivity analyses consistently found a negative correlation between PNI and DR prevalence, further supporting the reliability of our findings (Supplementary Table 1, Supplementary Tables 2, and Supplementary Table 3).

## Discussion

As far as we know, this is the first cross-sectional study using the NHANES database to analyze the relationship between PNI and DR. Using a nationally representative of diabetic adults from the United States, our findings demonstrated a notable inverse relationship between PNI levels and the prevalence of DR. After controlling for potential confounders, each unit increase in PNI corresponds to a 7% reduction in the prevalence of DR. In the subgroup analysis, we observed that hypertension significantly influenced the relationship between PNI and DR, with the negative correlation being more pronounced among individuals with hypertension. The results imply that individuals with lower PNI values may benefit from early interventions and targeted strategies aimed at enhancing nutritional health and mitigating inflammation, potentially reducing the incidence of DR.

PNI is a simple and readily accessible composite index with substantial clinical implications. Previous research highlights a strong link between PNI and onset, development, and chronic microvascular complications of diabetes. For instance, Zhang et al. conducted a cohort study exploring the association between PNI, all-cause mortality, and the emergence of diabetic nephropathy in individuals with T2DM. Their findings indicated that elevated PNI levels correlate with reduced risks of all-cause mortality and diabetic nephropathy^9^. Likewise, a cross-sectional study identified an inverse association between PNI and all-cause mortality among gestational diabetic patients^18^. Another cross-sectional study involving 128 patients with T2DM reported that lower PNI values are common in patients with DR^12^. These findings underscore PNI’s potential utility as a clinical marker for identifying diabetes-related vascular complications, including diabetic retinopathy.

PNI is derived from two key components: albumin and lymphocytes, which together provide an assessment of inflammation, nutritional status, and immune function. The specific physiological mechanisms that explain the connection between PNI and DR are not yet clearly understood and require additional research. However, several physiological mechanisms may explain the inverse association between PNI and DR.

First, serum albumin may delay the onset of DR due to its antioxidative and anti-inflammatory properties. Albumin, being the most prevalent serum protein produced by the liver, significantly contributes to minimizing oxidative stress and controlling inflammation—two critical factors in the development of diabetic retinopathy. Its antioxidant and anti-inflammatory properties may help protect retinal cells from damage and reduce the progression of vascular complications^19,20^. Excessive oxidative stress plays a pivotal role in the onset of insulin resistance, a fundamental process contributing to the development of diabetes. Serum albumin, with its potent antioxidant properties—such as its capacity for multiple ligand binding and trapping free radicals—may play a pivotal role in mitigating oxidative stress. These properties of serum albumin could be instrumental both the onset and development of diabetes, while also aiding in lowering the risk and slowing the progression of diabetic retinopathy, a common vascular complication of diabetes^21,22^. Lower serum albumin levels have been linked to elevated inflammatory markers^23^, with cytokines such as tumor necrosis factor-α (TNF-α) and interleukin-6 being closely associated with this reduction^24^. It is worth noting that normal physiological concentrations of albumin are capable of suppressing the TNF-α-induced expression of vascular cell adhesion molecules, thus mitigating inflammation. This anti-inflammatory property of serum albumin may play a protective role in vascular health, particularly in conditions like DR, where inflammation is a key pathological factor^25^. In recent decades, growing evidence has highlighted the critical roles of oxidative stress and chronic inflammation in the development of T2DM and DR. Oxidative stress can impair insulin signaling, promote vascular damage, and accelerate the progression of DR, while chronic inflammation contributes to endothelial dysfunction and retinal damage through the release of pro-inflammatory cytokines. Together, these mechanisms underline the importance of targeting oxidative stress and inflammation in the prevention and management of T2DM and its complications^26,27^. By reducing oxidative stress, trapping free radicals, and inhibiting the expression of pro-inflammatory cytokines and vascular adhesion molecules, albumin helps mitigate the inflammatory and oxidative processes that contribute to the vascular and retinal damage seen in DR. These protective properties suggest that maintaining optimal serum albumin levels could be beneficial in preventing or slowing the onset and development of DR.

Second, higher PNI may reflect lower levels of endothelial dysfunction, which contribute to retinal microvascular damage^28^. A cross-sectional study demonstrated that PNI levels are significantly inversely associated with albuminuria^5^. Albuminuria is a critical indicator of endothelial dysfunction, disrupting the delicate balance between vascular constrictors like angiotensin II and dilators such as nitric oxide. This disruption fosters vasoconstriction, modifies vascular permeability, and exacerbates endothelial impairment. Moreover, the presence of albumin in urine may trigger inflammatory responses in vascular endothelial cells, leading to apoptosis and weakening the endothelial barrier’s structural integrity^29–31^. The specific pathophysiological mechanisms involved still require further study.

DN and DR are both microvascular complications of diabetes. DN may affect serum albumin, an important factor in the calculation of PNI, thereby influencing the credibility of the negative association between PNI and DR. To further confirm the impact of DN on the observed negative association, we conducted subgroup analysis and interaction tests based on whether the participants had DN. The analysis results showed a stable negative association (OR < 1) between PNI and DR regardless of whether the participants had DN, and DN did not alter this association (*p* for interaction > 0.05). These analyses further validate the credibility of our findings.

The significant strengths of the study consist of its extensive and representative sample size, which improves the applicability of the results to larger populations. Additionally, the adjustment for potential confounding variables helps ensure that the observed associations are less likely to be influenced by other factors, thereby providing more accurate and reliable results. Furthermore, a subgroup analysis was conducted to evaluate the consistency of the relationship between PNI and DR across across different populations. These methodological strengths strengthen the study’s validity and support its conclusions regarding the relationship between PNI and DR prevalence. Our results underscore the potential role of maintaining high levels of PNI in preventing diabetic retinopathy, particularly among diabetic individuals with hypertension. This study further provides evidence supporting the potential role of controlling PNI through proper nutrition and inflammation control as a strategy to reduce the burden of DR.

There are a number of limitations that need to be taken into account when evaluating the findings. First, the cross-sectional study design measures exposure and outcome factors simultaneously, making it impossible to establish temporality and thus leaving the causal relationship between these factors uncertain. Secondly, our study relies on self-reported data and diagnostic codes from NHANES, which may introduce biases or inaccuracies. Thirdly, given the observational design of this study, the possibility of residual confounding from factors that were not measured cannot be excluded. Despite adjusting for numerous confounders, certain critical factors, including medications and conditions affecting albumin levels, were not controlled. These unmeasured variables might affect the observed association between PNI and albuminuria. Future longitudinal studies are warranted to ensure better control of confounding variables through improved experimental design and data analysis. Moreover, as a static indicator, PNI represents an individual’s current nutritional status but fails to capture its temporal changes. As PNI is subject to variation based on health conditions and lifestyle behaviors, a single measurement may fail to accurately represent long-term nutritional patterns or their effects on health. Future research should adopt large-scale longitudinal designs with multiple time-point data collection to better understand the dynamic relationship between PNI and DR.

Our findings highlight several directions for future research. First, longitudinal cohort studies are essential to determine the timing and progression relationship between PNI and DR. Second, clinical trials should investigate various strategies aimed at modulating PNI to prevent or manage DR. Third, exploring the underlying pathophysiological processes is vital for discovering new biomarkers and therapeutic targets.

## Conclusions

In conclusion, this research, based on a nationally representative cohort of diabetic adults in the United States, found an inverse association between PNI and the prevalence of DR. Compared to individuals with low PNI levels, the prevalence of DR is lower in those with high PNI levels, especially among individuals with hypertension. These findings suggest that maintaining an optimal PNI may play a role in preventing DR. Future longitudinal research is needed to verify the causal direction and uncover the mechanisms involved. Public health initiatives aimed at optimizing PNI levels could help mitigate the impact of DR.

## Electronic supplementary material

Below is the link to the electronic supplementary material.

Supplementary Material 1

## Data Availability

The original data presented in the study are openly available at https://www.cdc.gov/nchs/nhanes/index.htm (accessed in 2024). The data have not been previously presented orally or by poster at scientific meetings.

## Abbreviations

DR: Diabetic retinopathy
DN: Diabetic nephropathy
PNI: Prognostic nutritional index
T2DM: Type 2 diabetes mellitus
NHANES: National Health and Nutrition Examination Survey
NCHS: National Center for Health Statistics
PIR: Poverty-to-income ratio
TNF-α: Tumor necrosis factor-α
