# Supplementary material for: Association between prognostic nutritional index and diabetic retinopathy among U.S. diabetic adults in NHANES
Source: Sci Rep. 2025 Apr 15;15:12986. doi: 10.1038/s41598-025-96582-7 (PMC12000458; doi:10.1038/s41598-025-96582-7)
Supplement: Supplementary file 1 — Supplementary Material 1 [file 41598_2025_96582_MOESM1_ESM.docx]

**Supplementary Materials**

**Association Between Prognostic Nutritional Index and Diabetic Retinopathy among US Diabetic Adults: NHANES 2001-2018**

**Supplemental Table 1.** Unweighted logistic regression models for the association between prognostic nutritional index (PNI) and diabetic retinopathy (DR) in adults in the NHANES 2001–2018.

**Supplemental Table 2.** Weighted logistic regression models for the association between prognostic nutritional index (PNI) and diabetic retinopathy (DR) in adults with non-extreme PNI values in the NHANES 2001–2018.

**Supplemental Table 3.** Unweighted logistic regression models for the association between prognostic nutritional index (PNI) and diabetic retinopathy (DR) in adults with non-extreme PNI values in the NHANES 2001–2018.

**Supplemental Table 1.** Unweighted logistic regression models for the association between prognostic nutritional index (PNI) and diabetic retinopathy (DR) in adults in the NHANES 2001–2018.

| **Characteristic** | **Model 1 OR (95%CI)** | **Model 2 OR (95%CI)** | **Model 3 OR (95%CI)** |
| --- | --- | --- | --- |
| PNI | 0.93 (0.92,0.95) | 0.93 (0.91,0.95) | 0.93 (0.91,0.95) |
| **Categories** | | | |
| Q1 | 1.0 | 1.0 | 1.0 |
| Q2 | 0.87 (0.72, 1.06) | 0.86 (0.71, 1.05) | 0.87 (0.71, 1.06) |
| Q3 | 0.60 (0.50, 0.72) | 0.58 (0.48, 0.70) | 0.59 (0.49, 0.71) |
| Q4 | 0.53 (0.43, 0.65) | 0.50 (0.41, 0.62) | 0.51 (0.41, 0.63) |
| *p* for trend | <0.001 | <0.001 | <0.001 |

**Supplemental Table 2.** Weighted logistic regression models for the association between prognostic nutritional index (PNI) and diabetic retinopathy (DR) in adults with non-extreme PNI values in the NHANES 2001–2018.

| **Characteristic** | **Model 1 OR (95%CI)** | **Model 2 OR (95%CI)** | **Model 3 OR (95%CI)** |
| --- | --- | --- | --- |
| PNI | 0.91 (0.87,0.94) | 0.90 (0.87,0.94) | 0.90 (0.86,0.94) |
| **Categories** | | | |
| Q1 | 1.0 | 1.0 | 1.0 |
| Q2 | 0.96 (0.75, 1.22) | 0.96 (0.75, 1.23) | 0.92 (0.71, 1.19) |
| Q3 | 0.72 (0.52, 1.01) | 0.72 (0.51, 1.00) | 0.71 (0.51, 0.99) |
| Q4 | 0.51 (0.37, 0.69) | 0.50 (0.36, 0.68) | 0.48 (0.35, 0.66) |
| *p* for trend | <0.001 | <0.001 | <0.001 |

**Supplemental Table 3.** Unweighted logistic regression models for the association between prognostic nutritional index (PNI) and diabetic retinopathy (DR) in adults with non-extreme PNI values in the NHANES 2001–2018.

| **Characteristic** | **Model 1 OR (95%CI)** | **Model 2 OR (95%CI)** | **Model 3 OR (95%CI)** |
| --- | --- | --- | --- |
| PNI | 0.93 (0.90,0.96) | 0.92 (0.90,0.95) | 0.92 (0.90,0.95) |
| **Categories** | | | |
| Q1 | 1.0 | 1.0 | 1.0 |
| Q2 | 0.97 (0.78, 1.20) | 0.96 (0.78, 1.20) | 0.96 (0.77, 1.20) |
| Q3 | 0.68 (0.54, 0.84) | 0.66 (0.53, 0.83) | 0.67 (0.54, 0.84) |
| Q4 | 0.60 (0.48, 0.73) | 0.58 (0.47, 0.72) | 0.59 (0.47, 0.73) |
| *p* for trend | <0.001 | <0.001 | <0.001 |
